# Supplementary material for: Quantifying the Genetic Basis of Marfan Syndrome Clinical Variability
Source: Genes (Basel). 2020 May 20;11(5):574. doi: 10.3390/genes11050574 (PMC7288268; doi:10.3390/genes11050574)
Supplement: Supplementary file 1 [file genes-11-00574-s001.pdf]

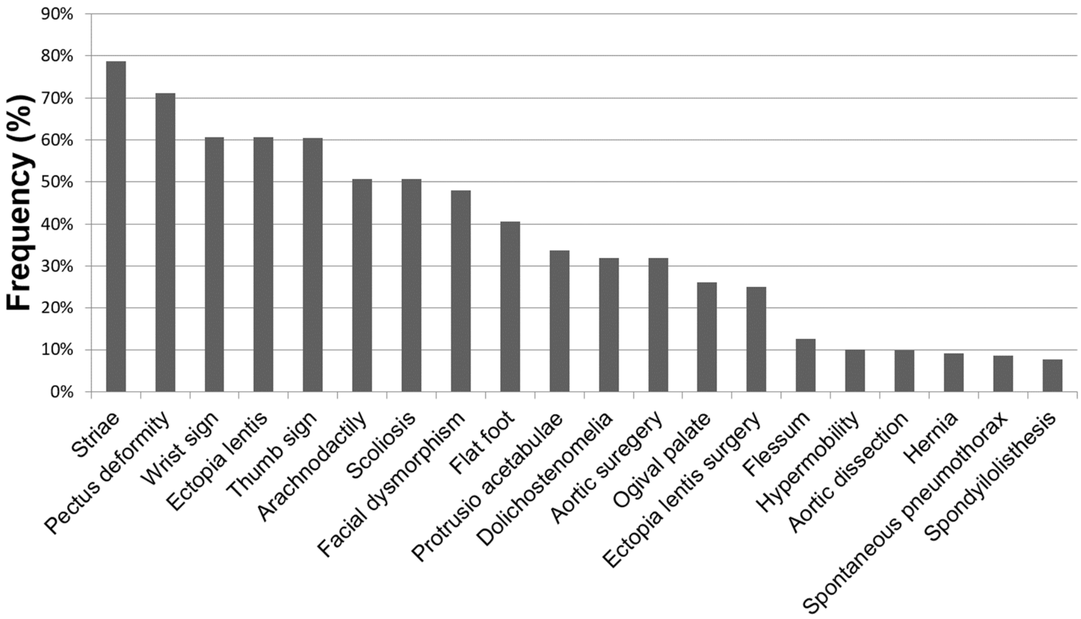

Figure S1: Frequency of MFS binary features across the study population

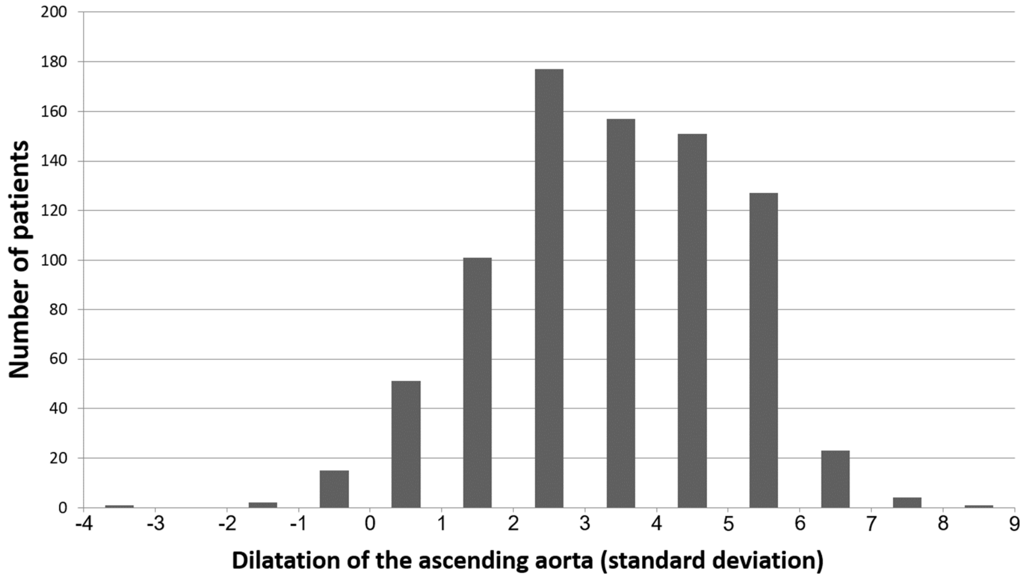

Figure S2: Distribution of the dilatation of the ascending aorta across the study population
Dilatation of the ascending aorta is expressed as Campens standard deviation.

$$Corr(Sib, AF) - Corr(Sib, PTC)$$

$$= \frac{Var(M_{spe}) + \frac{Var(A)}{2} + \frac{Var(D)}{4} + Cov(E)}{Var(M_{spe}) + Var(A) + Var(D) + Var(E)} - \frac{\frac{Var(A)}{2} + \frac{Var(D)}{4} + Cov(E)}{Var(A) + Var(D) + Var(E)}$$

$$= \frac{Var(M_{spe}) + \left(\frac{Var(A)}{2} + \frac{Var(D)}{4} + Cov(E)\right)\left(1 - \frac{Var(M_{spe}) + Var(A) + Var(D) + Var(E)}{Var(A) + Var(D) + Var(E)}\right)}{Var(M_{spe}) + Var(A) + Var(D) + Var(E)}$$

$$= \frac{Var(M_{spe}) - \left(\frac{Var(A)}{2} + \frac{Var(D)}{4} + Cov(E)\right)\left(\frac{Var(M_{spe})}{Var(A) + Var(D) + Var(E)}\right)}{Var(M_{spe}) + Var(A) + Var(D) + Var(E)}$$

$$= \frac{Var(M_{spe})\left(1 - \frac{\frac{Var(A)}{2} + \frac{Var(D)}{4} + Cov(E)}{Var(A) + Var(D) + Var(E)}\right)}{Var(M_{spe}) + Var(A) + Var(D) + Var(E)}$$

$$= H^2{}_{FBN1}[1 - Corr(Sib, PTC)]$$

Therefore:

$$H^2{}_{FBN1} = \frac{Corr(PO, AF) - Corr(PO, PTC)}{1 - Corr(PO, PTC)} \quad \text{(Equation 3)}$$

Similarly:

$$Corr(PO, AF) - Corr(PO, PTC)$$

$$= \frac{Var(M_{spe}) + \frac{Var(A)}{2} + Cov(E)}{Var(M_{spe}) + Var(A) + Var(E)} - \frac{\frac{Var(A)}{2} + Cov(E)}{Var(A) + Var(E)}$$

$$= H^2{}_{FBN1}[1 - Corr(PO, PTC)]$$

Therefore:

$$H^2{}_{FBN1} = \frac{Corr(PO, AF) - Corr(PO, PTC)}{1 - Corr(PO, PTC)} \quad \text{(Equation 3)}$$

Figure S3: Proof of Equations 2 and 3.

Corr: Pearson Correlation coefficient; Sib: Siblings; PO: Parent-offspring; AF: all *FBN1* families; PTC: subgroup of families with premature termination codon.

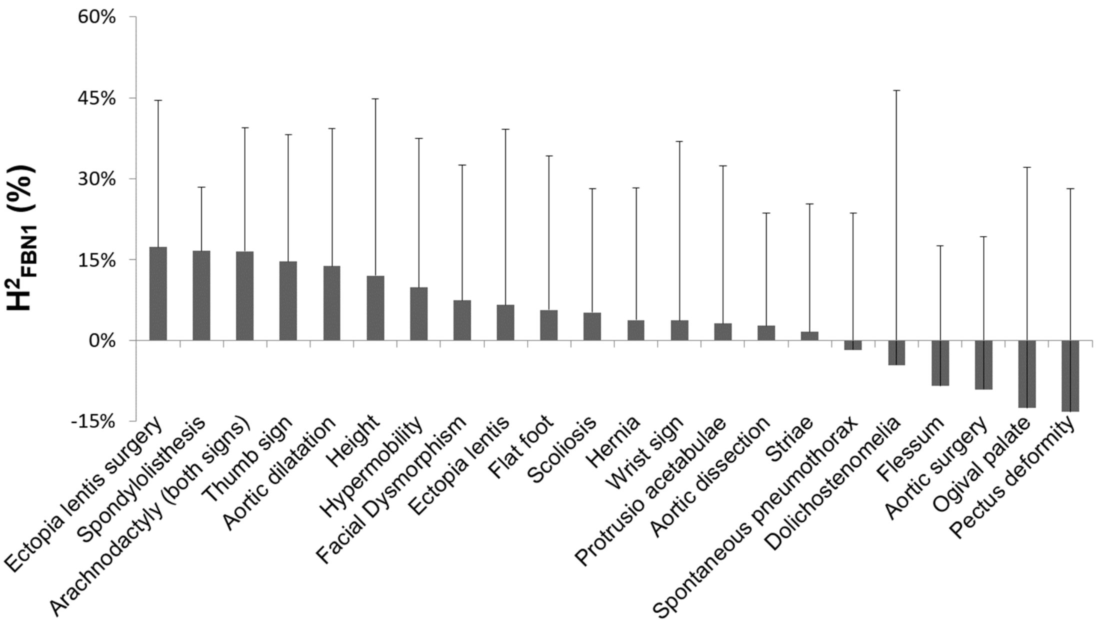

Figure S4: Estimation of the fraction of the phenotypic variance due to variations in the major locus *FBN1*

Comparison of phenotypic correlations between first degree relatives in all MFS families with respect to families with PTC mutations provided an estimate of the fraction of the phenotypic variance due to variations in the *FBN1* locus. SDs (represented by error bars) are quite high and prevented us from obtaining an accurate estimate for each clinical feature. However, 16 features out of 22 had a positive estimate (p=0.026), and the average estimate among features was only 4.4%, which reflected an overall significant though very low influence of the major locus.
